# Anti-listeria Activities of Linalool and Its Mechanism Revealed by Comparative Transcriptome Analysis

**DOI:** 10.3389/fmicb.2019.02947

**Published:** 2019-12-20

**Authors:** Zhipeng Gao, Joy D. Van Nostrand, Jizhong Zhou, Weiming Zhong, Kangyong Chen, Jiajing Guo

**Affiliations:** ^1^Hunan Engineering Technology Research Center of Featured Aquatic Resources Utilization, College of Animal Science and Technology, Hunan Agricultural University, Changsha, China; ^2^International Joint Lab on Fruits and Vegetables Processing, Quality and Safety, Hunan Key Lab of Fruits and Vegetables Storage, Processing, Quality and Safety, Hunan Agriculture Product Processing Institute, Hunan Academy of Agricultural Sciences, Changsha, China; ^3^Institute for Environmental Genomics, University of Oklahoma, Norman, OK, United States

**Keywords:** linalool, *Listeria monocytogenes*, antimicrobial, anti-biofilm, transcriptome

## Abstract

*Listeria monocytogenes*, which causes serious foodborne infections and public health problems worldwide, is one of the most important foodborne pathogens. Linalool has been identified as an antimicrobial agent against some microorganism, but its mechanism of action is currently unclear. Here, we investigated the efficacy of linalool against *L. monocytogenes* while planktonic and as a biofilm and explored potential mechanisms of action. Linalool exhibited strong anti-listeria activity in the planktonic stage. Scanning electron microscopy (SEM) and transmission electron microscopy (TEM) observations revealed seven stages were classified of cells at microscopic level. Mesosome-like structures were observed for the first time in *L. monocytogenes* after linalool treatment. Linalool also showed significant anti-biofilm activity through both dispersal and killing of cells in the biofilm based on confocal scanning laser microscopy (CLSM) and SEM imaging, crystal violet staining, XTT and COMSTAT assays. Moreover, comparative transcriptome analysis demonstrated many potential mechanisms of action for linalool and some important pathways were screened out through the analysis of GO enrichment and KEGG. Our study provides evidence that linalool exhibits a strong antimicrobial activity against both the planktonic and biofilm forms of *L. monocytogenes* and gives insight into its mechanism of action.

## Introduction

*Listeria monocytogenes* (*L. monocytogenes*) is a Gram-positive pathogen that is able to survive under a variety of adverse environmental conditions such as refrigeration temperatures, low pH, hyperosmotic and high salt environments (Gandhi and Chikindas, 2007). It is widely distributed in nature and can cause listeriosis, a severe food-borne zoonosis by contaminating ready-to-eat food, dairy products, undercooked food and food processing equipment (Buchanan et al., 2017). Listeriosis may result in enterogastritis, meningitis, septicemia, hemorrhagic rash and mononucleosis with a high mortality rate, especially in neonates, pregnant women, the elderly, and persons with immunodeficiency^1^. Hence *L*. *monocytogenes* has gained widespread attention worldwide because of its risks to human safety.

*Listeria monocytogenes* has the ability to form biofilms by attaching to different material surfaces, such as stainless steel, plastic, glass, rubber, and polymers (Møretrø and Langsrud, 2004). Biofilms are defined as highly organized bacterial communities adhering to biotic or abiotic surfaces and embedded in extracellular polymeric substances (O’Toole et al., 2000). Compared to planktonic cells, biofilms are more resistant to antimicrobials and can have an advantage in gaining nutrients if there is strong competition between planktonic and biofilm cells (Kragh et al., 2016). *L. monocytogenes* biofilms are more difficult to eradicate from contaminated food than planktonic cells and have a greater potential to infect humans and lead to listeriosis than planktonic cells. Thus, multipronged strategies are needed to prevent or eradicate *L. monocytogenes* biofilm.

In the food industry, synthetic chemical preservatives are often used to control food-borne pathogens and spoilage, but they may have harmful side-effects to human health, such as allergies, neurological damage and other potential harms (Furrer et al., 2002; Soni et al., 2005; Anand and Sati, 2013). Therefore, there is a need for broad-spectrum, high-efficiency and non-toxic natural preservatives. In recent years, essential oils, volatile aromatic oily liquids extracted from plants, have attracted attention for their antimicrobial properties (Bajpai et al., 2013; Basak and Guha, 2017; Kumari et al., 2017; Manoharan et al., 2017). But the use of essential oils by the food industry has been limited due to the high volatility of the oils and inconsistent chemical composition from differences in harvest season, growing conditions and geographical regions (Hussain et al., 2008; Huang et al., 2009; Settanni et al., 2014). Thus isolating the specific essential oil component exhibiting antimicrobial properties may be a good way around this problem.

Linalool (3,7-dimethyl-1,6-octadien-3-ol) is an acyclic monoterpene tertiary alcohol with volatile flavors found in numerous plant essential oils. Linalool is usually used as a food additive and is considered to be generally recognized as safe (GRAS)^2^. Linalool has different biological properties including sedative (Guzmán-Gutiérrez et al., 2012), anxiolytic (Linck et al., 2010), anticonvulsant (Elisabetsky et al., 1999), anesthetic (Narusuye et al., 2005), analgesic (Batista et al., 2010), anti-inflammatory (Huo et al., 2013), antioxidant (Atsumi and Tonosaki, 2007), and antimicrobial (Park et al., 2012) activities. Compared to its other biological properties, studies on the antimicrobial activity of linalool are limited. We previously demonstrated that linalool from citrus essential oils has strong antimicrobial properties (Guo et al., 2018). The aim of this study was to evaluate the antibacterial activity and unravel potential mechanisms of action against *L. monocytogenes*. To the best of our knowledge, this is the first study to estimate the anti-biofilm activity of linalool against *L. monocytogenes*, and also the first time RNA-seq analysis was used to explore the antimicrobial mechanism of linalool.

## Materials and Methods

### Bacterial Strains

*Listeria monocytogenes* (ATCC 19115) was obtained from Guangdong Microbiology Culture Center (GMCC, Guangdong, China) and stored at −80°C.

### Linalool

Linalool (95%) was purchased from Sigma-Aldrich (United States). The antimicrobial activity of linalool was determined in our previous study, and the MIC value was 0.5% (v/v) (Guo et al., 2018).

### Growth Experiments

To culture planktonic *L*. *monocytogenes*, the bacterial suspension were grown up overnight using Brain Heart Infusion broth (BHI, Guangdong Huankai Microbial, China) at 37°C with shaking. Linalool was added to the bacterial suspensions (1 × 10^7^ CFU/ml) at 1 × MIC (0.5% v/v or 4.35 mg/ml); no linalool was added to the positive control. Tween 20 (1% v/v final concentration) was then added to all samples to increase solubility and then the samples were incubated at 37°C. At 4, 8, and 12 h, bacterial suspensions were harvested by centrifugation at 4000 rpm for 10 min and then carefully washed three times with PBS. The pellets were processed immediately for SEM and TEM assay. For RNA-sequencing, pellets were kept at −80°C till RNA extraction.

To form mature biofilm of *L*. *monocytogenes*, the bacterial suspension was cultured overnight at 37°C, then 2000 μl bacteria suspension were added to each well (with embedded coverslips) in 24-well tissue culture plates and incubated at 37°C for 24 h, then the fresh broth was added to each well after remove the old broth, and incubated for 24 h, and then the broth was changed again and incubated for another 24 h. After incubated for 72 h in total, 2000 μl fresh broth containing linalool (1 × MIC concentration) was added to each well, then the plates were incubated for a series of time intervals (8, 16, and 24 h) at 37°C, the broth without linalool added was used as positive control (Guo et al., 2019). After incubation, the old broth were removed and the samples were washed with PBS three times. Then the coverslips with biofilm attached were processed immediately for crystal violet staining, live dead staining, XTT and SEM assay.

### Microscopy

#### Scanning and Transmission Electron Microscopy (SEM and TEM)

For EM work, the cells were fixed in 2.5% glutaraldehyde overnight at 4°C and washed again with PBS three times. For SEM, the bacterial cells were then dehydrated at room temperature with a gradient concentration of ethanol (10, 30, 50, 70, 90, and 100%) for 15 min at each concentration. The prepared samples were then freeze-dried, coated with gold film and visualized by Scanning Electron Microscope (Hitachi SU8010). For TEM, the cells were negatively stained with 1% phosphotungstic acid for 5 min, and finally examined by Transmission Electron Microscope (Hitachi HT-7700).

#### Live Dead Staining

Treated biofilm samples were fixed in 4% paraformaldehyde (PFA) for 4 h at 4°C, then stained with a bacterial live/dead stain using the Filmtracer^TM^ LIVE/DEAD^TM^ Biofilm Viability Kit (FilmTracer^TM^, Molecular Probes^®^, Thermo Fisher, United States) in the dark at room temperature for 30 min, then gently washed with PBS three times to remove the stain. The samples were imaged with a CLSM (Zeiss LSM880), using a 63 × /1.40 oil objective at 488 nm excitation. COMSTAT 2 software was used to evaluate the biomass, average thickness and the surface volume ratio of the biofilm (Heydorn et al., 2000).

### Crystal Violet Staining Assay (CV)

The ability of linalool to eradicate *L. monocytogenes* biofilms was measured by CV assay (Gao et al., 2015). Treated biofilm samples were washed with PBS three times and fixed with methanol at room temperature for 30 min, air-dried, then 200 μl of 0.1% crystal violet dye was added to each well. After 30 min at room temperature, the samples were washed with PBS three times, air-dried again, then 200 μl of 95% ethanol was added to release the bound dye. The absorbance was then measured using microplate reader at 630 nm.

### Biofilm Metabolic Activity (XTT Reduction Assay)

The XTT reduction assay was carried out using a Cell Proliferation Kit II (XTT) (Sigma- Aldrich) (Sivaranjani et al., 2016). Briefly, 100 μl PBS and 50 μl XTT regent were added to each well and incubated for 24 h at 37°C in the dark. The absorbance was then measured by a microplate reader at 450 nm.

### RNA-Seq and Bioinformatics Analysis

Total RNA was isolated following the manufacturer’s protocol of Majorbio company (Shanghai, China). RNA quality was assessed using the OD_260_ nm/OD_280_ nm ratio as measured on a Nanodrop 2000 (ThermoFisher, United States). All samples were between 1.8 and 2.0. Before library construction, rRNA was removed with the Ribo-Zero rRNA Removal Kit (Epicentre, San Diego, CA, United States) and mRNAs were fragmented into ∼200 bp fragments using metal ions. The mRNA fragments were then transcribed into first-strand cDNA using reverse transcriptase followed by second strand cDNA synthesis. Libraries were constructed by digesting the second strand cDNA with UNG enzyme.

Sequencing data was subjected to quality control and low quality reads were removed. Raw reads were filtered into clean reads and subjected to subsequent bioinformatics analysis, all the subsequent analyses were performed using the clean data. The datasets generated for this study can be found at NCBI, GSE136998.

### Quantitative Real-Time Polymerase Chain Reaction (qRT-PCR) Analysis

Isolation of total RNA from *L. monocytogenes* was carried out using Trizol following the manufacturer’s instructions. A HiScriptTM Q RT SuperMix for qPCR (Vazyme, Nanjing, China) was used for the synthesis of cDNAs, and qRT-PCR was performed using AceQTM qPCR SYBR^®^ Green Master Mix (Vazyme, China). Forward and reverse primers are listed in Table 1. qRT-PCR was carried out on an Applied Biosystems 7300 Real Time PCR System using the following program: 95°C for 5 min, 40 cycles of 95°C for 5 s, 55°C for 30 s and 72°C for 40 s. Gene expression levels were normalized using the housekeeping gene rpoB as a reference. The relative expression levels of genes were presented as 2−ΔΔCT values. All samples were analyzed in triplicate, and the mean values were used for calculating mRNA levels.

**TABLE 1:** Oligonucleotide primers used for qRT-PCR analysis.

Gene	Primer name	Oligonucleotide (5′-3′)
*Ffh*	*Ffh*-F	ATGGCATTTGAAGGACTA
	*Ffh*-R	CACGTTCGCTTACTGTTT
*dltA*	*dltA*-F	CTTGGATGTATGGGTTTC
	*dltA*-R	CTGTAGGTCCGTATGTATT
*dltD*	*dltD*-F	AAGGTTTCCGTTACTACTC
	*dltD*-R	CTGCTAATACTTGGTCGA
*dltB*	*dltB*-F	TATGACGCTTTCTTTCTG
	*dltB*-R	ATATGCTAGGTAGGCAAT
*htrA*	*htrA*-F	TGTTATTATCGGAGGACTT
	*htrA*-R	TACACTGACTACCGCATC
*secG*	*secG*-F	CGGTCTTACTCATCATCG
	*secG*-R	AATAATTGCTCAGCTCCA
*cydA*	*cydA*-F	TTGGAGCTTCCGTATCAT
	*cydA*-R	TGTCGCCCTATTTCTGTC
*rpoB*	*rpoB*-F	CGTCGTCTTCGTTCTGTTGG
	*rpoB*-R	GTTCACGAACCACACGTTCC

### Statistical Analysis

All experiments were carried out in triplicate. The Student’s *t*-test was used for statistical analysis using GraphPad Prism 6 software. Significant differences (*P* < 0.05) are indicated by asterisks in all figures.

## Results

### Anti-planktonic Cells and Anti-biofilm Activity

The *in vitro* antibacterial efficacy of linalool against planktonic *L. monocytogenes* cells was evaluated in our previous study (Guo et al., 2018). In that study, fourteen species of citrus EOs were tested for their antimicrobial activity, and seven major components were screened out based on our statistical analysis. Linalool is the one showed the greatest effect against *L. monocytogenes*, and the ZOI, MIC and MBC values were 39.58 ± 0.74mm, 0.5% (v/v) and 1% (v/v), respectively.

**FIGURE 1:**
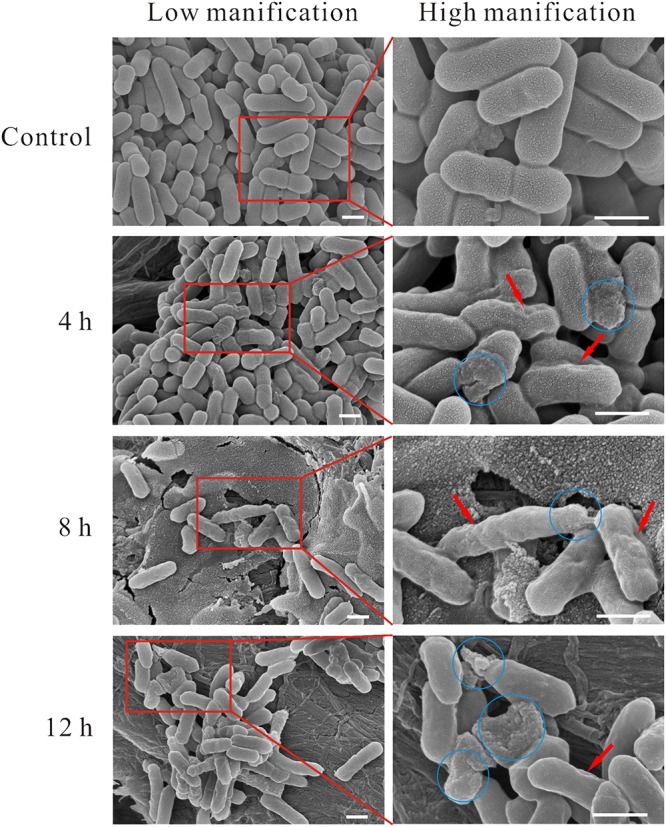
SEM micrograph of *L. monocytogenes* planktonic cell treated and untreated with linalool. (Control) untreated bacteria; (4, 8, 12 h) bacteria treated with linalool at 1 × MIC for 4, 8, and 12 h respectively. Images on the right side are the magnification of the red rectangle in the left side. The red arrows indicated the wrinkled surface of cells, the blue circles indicated the lysis of cells, surface collapse and cell fragment. The scale bar represented 500 nm.

Morphological changes of linalool-treated planktonic cells were observed using SEM. As shown in Figure 1, bacterial cells without linalool treatment (control) were rod-like with a size ranged of 1–2 μm × 0.5 μm (length × width), the surface of the cells were intact and smooth. After linalool exposure, while the size of the cells was not obviously changed, some morphological changes were observed. The cell surface was wrinkled (shown with the red arrows) at all time-points, but the percentage of affected cells increased with exposure time. Cell lysis, surface collapse and cell fragments were also observed (indicated by the blue circle) in all three treated groups, but complete lysis was often observed at 12 h.

**FIGURE 2:**
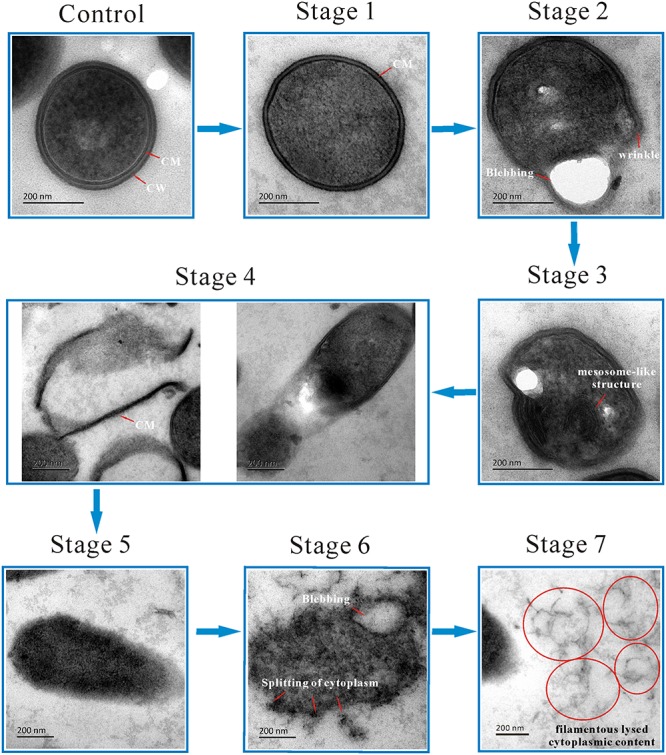
TEM micrograph showing the morphological changing process of *L. monocytogenes* planktonic cell after treated with linalool. (Control) untreated bacteria; (Stage 1–7) bacteria treated with linalool. The red arrows and circles (together with white characters beside them) indicated the structure or the morphological changes of cells. CM and CW represented cytoplasmic membrane and cell wall respectively.

Next, cells were visualized using TEM to examine the cellular microstructure of *L. monocytogenes* after exposure to linalool (Figure 2). Control group cells were intact with a homogeneously distributed cytoplasm and displayed a heterogeneous electron density. The cell wall (CW) and cytoplasmic membrane (CM) were well-defined and could be identified easily. Obvious morphological changes were observed in linalool treated cells. Seven stages of morphological change were identified. In Stage 1, the cell had lost its CW but had an intact CM. In Stage 2, the CM began to form wrinkles along the surface, as was also observed in SEM images, and blebs or vacuoles had formed. The cytoplasm had also become heterogeneous and had “bare spots.” In Stage 3, mesosome-like structures (MS) were observed in the cytoplasm. A further description of these structures is below. In Stage 4, part of the CM had been ruptured causing a loss of cytoplasm and some empty CMs were evident. In Stage 5, the amount of CM remaining was greatly reduced and in some cases was completely missing, although the cytoplasm remained intact. In Stage 6, without the protection of CM, cytoplasm had begun to separate, but cell shapes could still be easily identified. Finally, in Stage 7, the cell had completely lysed and filamentous cytoplasm was observed, but without a cell-like shape. Although we identified seven stages of cell lysis, this is likely a continuous process and some stages may occur concurrently.

**FIGURE 3:**
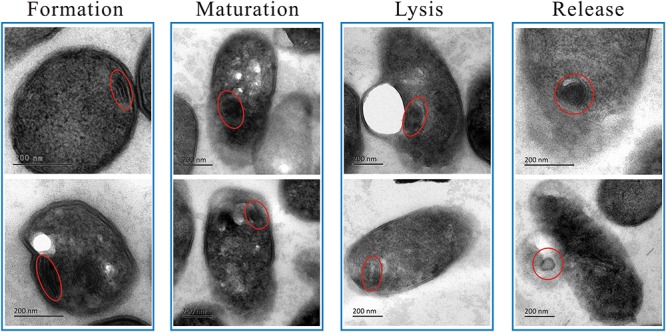
TEM micrograph showing the four changing stages of mesosome-like structures of *L. monocytogenes* planktonic cell after treated with linalool. The red circles indicated the mesosome-like structures.

To the best of our knowledge, this is the first report of MS in *L. monocytogenes* cells after linalool treatment, so these structures were examined in more detail (Figure 2, Stage 3; Figure 3). The MS were often observed at 4 h but rarely at 8 or 12 h. When present, there were 1–2 MS per cell but most only had 1. The MS were multi-lamellar (3–5 lamellas), but not multi-reticulated, with an average diameter of 100–150 nm. Based on our observations, there were four stages of MS formation: “Formation,” “Maturation,” “Lysis” and “Release” (Figure 3). During formation, some membrane-like structures were present near the CM, particularly at wrinkled areas. These structures were linear rather than circular. During maturation, multi-lamellar structure appeared in the cytoplasm. During lysis, the membrane-like structures were destroyed. As a result, the number of lamellas decreased and they were no longer completely intact, but had some gaps or holes. At the release stage, the cell integrity had weakened, releasing some of the cytoplasmic content, including these structures.

**FIGURE 4:**
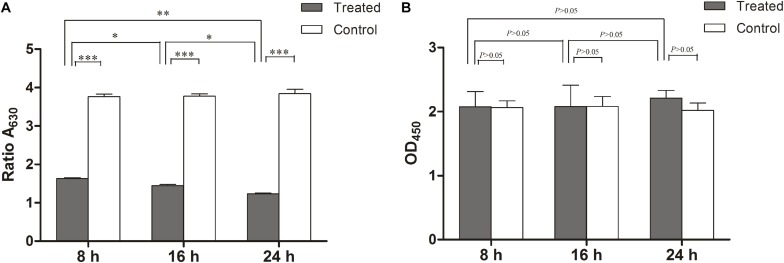
Anti-biofilm activity of linalool against 72 h preformed biofilm of *L. monocytogenes*. **(A)** Effect of linalool on the growth and development of biofilm in control, 8, 16, and 24 h treatment groups, as determined by the CV assay. **(B)** Effect of linalool on the metabolic activity of biofilm in control, 8, 16, and 24 h treatment groups, as determined by the XTT assay. Bars represented the mean values (Mean ± SD; *n* = 3). “^∗^” pointed to significantly enrichment. *P* < 0.001 were labeled as “^∗∗∗^”, *P* < 0.01 were labeled as “^∗∗^”, and *P* < 0.05 were labeled as “^∗^”.

Since biofilms are more resistant to antimicrobials compared to planktonic cells, *L. monocytogenes* can form biofilms on the surfaces of food processing equipment, presenting a health hazard in the food industry (Djordjevic et al., 2002; Srey et al., 2013). So, in this study we also examined the anti-biofilm activity of linalool against *L. monocytogenes*. Biofilms were treated with linalool (1 × MIC) at 8, 16, and 24 h. Linalool was able to reduce the biofilm biomass over time, with a reduction of 56.6, 61.7, and 67.3% at 8, 16, and 24 h, respectively (Figure 4A). As shown in Figure 4B, there were no difference of the metabolic activity of the biofilms with or without linalool treatment or among any of the time-points examined.

**FIGURE 5:**
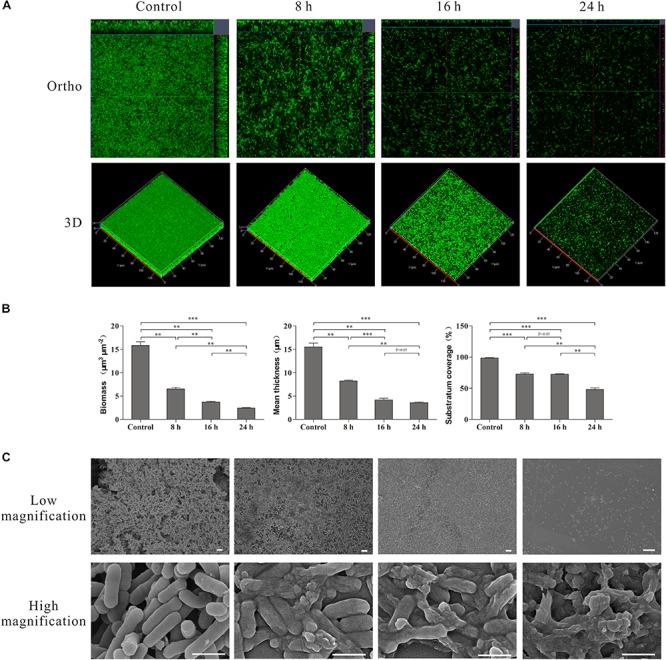
CLSM, SEM images and COMSTAT analysis of the *L. monocytogenes* biofilms untreated (Control) and treated by linalool for different time (8, 16, and 24 h). **(A)** CLSM photography. **(B)** COMSTAT analysis. Bars represented the mean values (Mean ± SD; *n* = 3). **(C)** SEM photography. The scale bar represented 500 nm.

The effect of linalool on the biofilms and individual cells was further examined using CLSM with Live/Dead staining and SEM analysis. The images acquired by CLSM are shown in Figure 5A. The control cells exhibited a mature biofilm with intact multilayered and three-dimensional structures. But a significant loss of biomass, mean thickness and substratum coverage was observed over time after treatment with linalool (Figures 5A,B). Hollows and holes in the biofilm were obvious after treatment and the mean thickness of the biofilm reduced over time until only a thin monolayer of cells remained at 24 h.

SEM was used to examine morphological changes of individual cells (Figure 5C). At a low magnification, the control group cells aggregated together to form macro-colonies and a mature biofilm was evident with an intact and complex structure. After treatment with linalool, the biofilm structure was disrupted and only some micro-colonies were observed after 8 and 16 h. By 24 h, only single cells were present. At high magnification, all cells were intact in the control. Rod-like cell aggregates and cell division were observed, indicating the cells within the biofilm were growing and cell reproduction was occurring. After treatment, lysed and dead cells were apparent. The percentage of lytic cells increased over time, with cell fragments and very few intact cells obvious at 24 h.

### Transcriptomic Analysis

**FIGURE 6:**
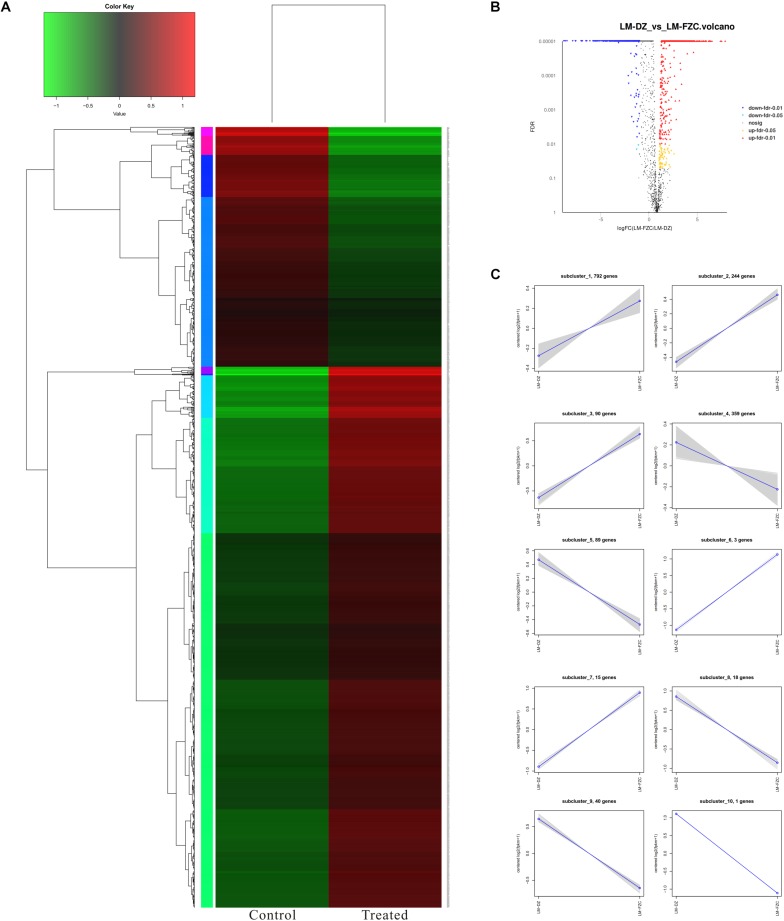
Differentially expressed genes (DEGs) analyzed in *L. monocytogenes* planktonic cells between treated group (treated with linalool) and control group (untreated with linalool). **(A)** Heat map of DEGs. Red cluster indicated up-regulated genes and blue cluster indicated down-regulated genes. **(B)** Volcano plot of DEGs. Percentages of up-regulated and down-regulated genes for each group indicated on a volcano plot. **(C)** log2 (ratio) line chart. Gray lines were relative expressions of genes in different groups. Blue lines were average values of relative expressions of genes in different groups.

RNA-Seq was used to unravel the potential antimicrobial mechanism of linalool. Data from control *L. monocytogenes* planktonic cells and those treated with linalool for 8 h is shown in Figure 6. A total 2824 genes [1897 genes down-regulated (≤-2-fold) and 927 genes up-regulated (≥2-fold)] were differentially expressed by the treated cells.

**FIGURE 7:**
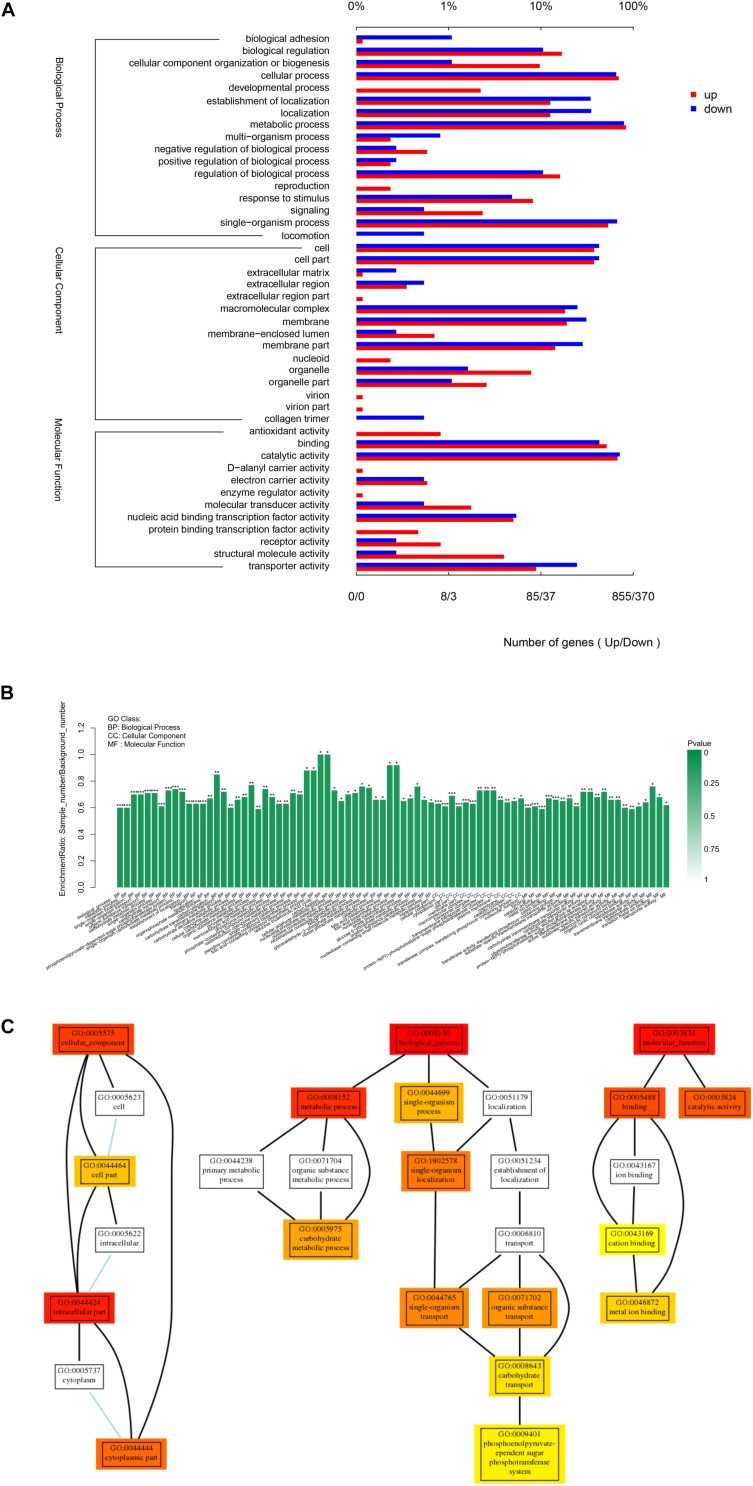
Gene Ontology (GO) analysis of differentially expressed genes. **(A)** GO classification of DEGs. **(B)** GO enrichment of DEGs. Genes were annotated in three main categories: biological process, cellular component and molecular function. **(C)** Go hierarchical structure. The three sub-graphs represented the three categories of GO as mentioned above, each rectangle represented a GO term. The colored rectangle represented the GO term with significant difference, the closer the color was to red, the more significant the difference. The line between GO terms represented the relationship between two GOs. “^∗^” Indicated significantly enrichment. *P* < 0.001 were labeled as “^∗∗∗^”, *P* < 0.01 were labeled as “^∗∗^”, and *P* < 0.05 were labeled as “^∗^”.

The Gene Ontology (GO) classification of the genes that were up- and down-regulated in the treated cells was shown in Figure 7A. All the DEGs (Differentially Expressed Genes) were classified into forty-four GO categories. Of these, 17 categories were related to biological processes and the largest number of DEGs were for metabolic, cellular and single-organism processes. Fifteen categories were related to cellular components, with the largest three for cell parts, membrane parts and macromolecular complexes. Twelve categories were related to molecular functions and the largest number of genes were for catalytic activity, binding and transporter activity.

In order to better visualize the results, the level of enrichment and hierarchical structure of GO groups were examined (Figures 7B,C). Compared with the control, the treatment groups had significantly different level of expression for genes relevant to cellular components (GO:0005575), especially intracellular (GO:0044424), cytoplasmic (GO:0044444), and cell components (GO:0044464), which suggests that exposure to linalool caused changes to the cell structure and to cellular components both inside the cell and on the surface of the cell. Many genes involved in biological process (GO:0008150) altered. Metabolic process (GO:0008152) and especially carbohydrate metabolic process (GO:0005975) were influenced by the treatment of linalool. Meanwhile, single-organism process (GO:0044699) especially single- organism localization (GO:1902578), single-organism transport (GO:0044765) and carbohydrate transport (GO:0008643) were impacted significantly. Numerous genes related to molecular function (GO:0003674) changed, especially binding (GO:0005488) and catalytic activity (GO:0003824), and when focused on binding, cation binding (GO:0043169) and metal ion binding (GO:0046872) are the most two variable GO terms.

**FIGURE 8:**
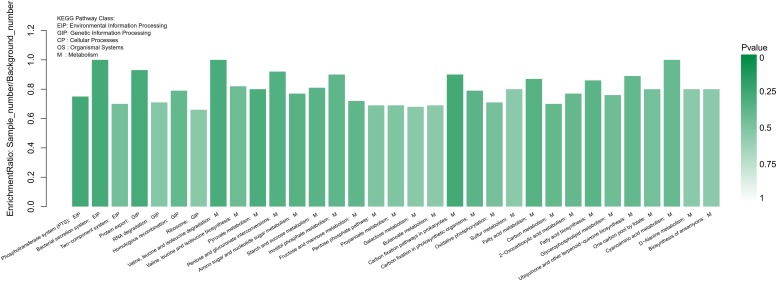
Statistical enrichment of differentially expressed genes in KEGG pathways. Genes were annotated in five main categories: Environmental Information Processing (EIP) pathway, Genetic Information Processing (GIP) pathway, Cellular Processing (CP) pathway, Organismal Systems (OS) pathway and Metabolism (M) pathway. The green color represented the *p*-value as shown on the right side, the darker color meant the more significant.

Finding the changes of biological functions are a key way to unravel the mechanism of antimicrobial activity. Here, KEGG (Kyoto Encyclopedia of Genes and Genomes) pathway enrichment analysis was carried out to study the biological functions of genes at the molecular, cellular and organism levels (Figure 8). Thirty-three pathways were significantly enriched. Of these, three were from environmental information processing (EIP) pathways, four were from genetic information processing (GIP) pathways and 26 were from metabolism (M) pathways. Nearly 79% of the pathways with significant differences in expression level were related to metabolism, which suggested that the mechanism of action may involve metabolic changes to the cell. KEGG annotation was also carried out. In total, 150 KEGG annotation figures were found.

### Validation of DEGs by qRT-PCR

**FIGURE 9:**
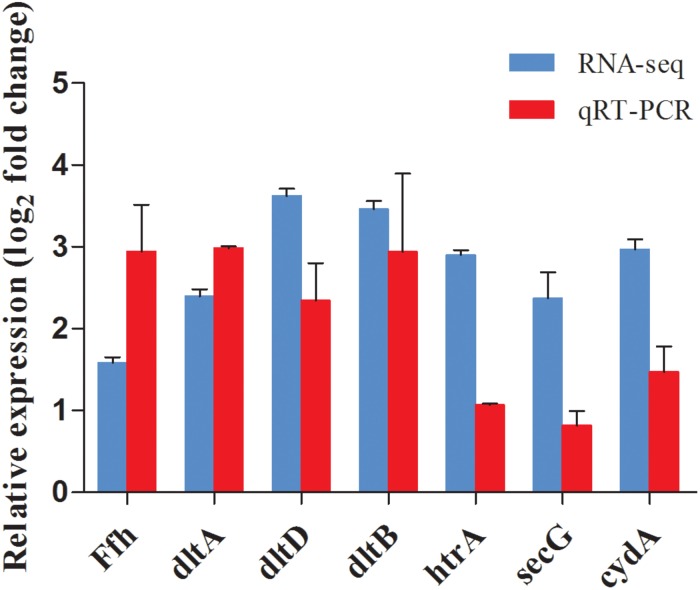
Comparison of gene expression values between RNA-Seq and qRT-PCR analysis. Bars represented the mean values (Mean ± SD; *n* = 3). Gene expression levels were normalized using the housekeeping gene rpoB as a reference.

To validate the reliability of the RNA-Seq results, seven DEGs (shown in Table 1) were selected for qRT-PCR analysis. Similar trends were observed between qRT-PCR and transcriptome data, indicating the RNA-Seq data was reliable (Figure 9).

## Discussion

Studies regarding the anti-planktonic and anti-biofilm activity of linalool are limited and to the best of our knowledge there are no published studies on the anti-biofilm effects of linalool against *L. monocytogenes*. Mith et al. (2014) investigated the antimicrobial efficacy of linalool against some food-borne and food-spoilage bacterial pathogens. They examined two *L*. *monocytogenes* strains (*L*. *monocytogenes* NCTC 11994 and *L*. *monocytogenes* S0580) and found a ZOI of 11.4 ± 0.1 mm and 12.8 ± 0.3 mm, respectively (Mith et al., 2014). We observed a much larger ZOI (39.58 ± 0.74 mm) in the current study, which could be due to differences in the bacterial strains used. The antimicrobial effect of linalool against periodontopathic and cariogenic bacteria was studied by Park et al. (2012). The results showed that linalool exhibited remarkable antibacterial effects against these bacteria, with MIC and MBC values ranging from 0.1 to 1.6 mg/ml (Park et al., 2012). Duarte et al. (2016) evaluated the potential usage of linalool to control *Campylobacter jejuni* and *Campylobacter coli*, the growth of these two strains was strongly inhibited by linalool. The DIZ was more than 85 mm and the MIC values were 0.5–1 ml/ml (Duarte et al., 2016). Aelenei et al. (2019) studied the antibacterial activity of linalool against some Gram-positive (methicillin-susceptible and methicillin-resistant *Staphylococcus aureus*, MRSA; *Staphylococcus epidermidis*) and Gram-negative bacteria (*Pseudomonas aeruginosa*, *Escherichia coli*) using broth microdilution assays. MICs of 5.36 μg/ml were observed for all of these bacteria (Aelenei et al., 2019). Bagamboula et al. (2004) investigated the inhibitory effect of linalool against *Shigella sonnei* and *Shigella flexneri* using an agar diffusion method. The inhibition zones ranged from 0 to 4 mm and linalool showed a limited antibacterial activity against these bacteria (Bagamboula et al., 2004). Another study found a linalool MIC of 0.125% for *S*. *flexneri* (Ngome et al., 2018). The efficacy of linalool varied for different bacterial species and strains.

While not tested with *L. monocytogenes*, the effect of linalool on biofilms has been examined for some bacteria. Ngome et al. (2018) observed a minimum bactericidal concentration of 3% against *S*. *flexneri* biofilms. Linalool was able to inhibit the biofilm formation against *Candida albicans* (Hsu et al., 2013), but did not significantly affect *P*. *aeruginosa* or Enterohemorrhagic *E*. *coli* (EHEC) (Kim et al., 2015). As with the studies of planktonic cells, results from studies of biofilms indicated the efficacy of linalool varies between bacterial species of microorganism, although for some bacterial or fungal strains linalool would be a good candidate to inhibit or eradicate biofilms. While linalool was able to disrupt biofilms, some biofilm still remained even after 24 h. A higher dosage may be required to completely eliminate the biofilm. The cellular biomass decreased significantly between control and 8 h treatment, but the declining trend slowed down after 16 and 24 h treatment, which implied that dispersal might be a main effect at earlier stage of treatment. Meanwhile, the high proportion of lysed and dead cells in 16 and 24 h treatment indicated that killing of cells might be a main effect at later stage of treatment.

Mesosomes are membranous structures present in both gram-positive and gram-negative bacteria and are involved in synthesis of the CW and the CM, cell division, cell septum formation, separation and replication of nucleoids, enzyme transport (Salton, 1967; Greenawalt and Whiteside, 1975; Li et al., 2017). Recently, more and more studies have shown the presence of mesosomes in bacteria cells after treatment with antimicrobial drugs, suggesting mesosomes may be important to bacteria for responding to and surviving exposure to antibiotics. Several antibiotic efficacy studies have been performed with *S*. *aureus* and/or MRSA, including retinoids (Kim et al., 2018), violacein (Aruldass et al., 2018), defensins (Shimoda et al., 1995), trimethoprim (Nishino et al., 1987), rifampin (Gottfredsson et al., 1993), cationic peptides (Friedrich et al., 2000), tetrabromobisphenol A (Wang et al., 2018), antimicrobial peptides (Hartmann et al., 2010), photodynamic therapy (Iluz et al., 2018), porphyrins (Malik et al., 1988), silver nanoparticles (Krychowiak et al., 2018). In addition, imipenem has been studies with *P*. *aeruginosa* (Gottfredsson et al., 1993) and rifampicin has been studies with *Xanthomonas campestris* (Li et al., 2008) and *E*. *coli* (Xin et al., 2014). In all of these studies, the antibiotic induced the presence of mesosomes. Based on our results and those from previous studies, the mesosomes may be due to an alteration of CM, which also suggests that the CM may be an important target for linalool. The “life-cycle” stages of the mesosomes we observed (Figure 3) suggest that the formation of mesosomes is not instantaneous. As such, linalool might affect synthesis of the CM. Furthermore, since the CM is crucial for synthesis of the CW, the alteration of the CM may also affect the CW integrity.

Several genes related to peptidoglycan biosynthesis were up-regulated. The *mur* operon (*murA*, *murB*, *murC*, and *murD*) were significantly up-regulated. As shown in Supplementary Figure S1, MurA firstly transfers enolpyruvyl moiety to uridine diphosphate (UDP)-*N*-acetylglucosamine (GlcNAc), to generate enolpyruvyl UDP-GlcNAc. Then MurB catalyzes enolpyruvyl UDP-GlcNAc to UDP-MurNAc, and MurC catalyzes the addition of L-Ala onto UDP-MurNAc. Finally, MurD catalyzes the addition of D-Glu to UDP-MurNAc-LAla. Meanwhile, MraY, involving in the transfer of MurNAc- pentapeptide onto undecaprenyl phosphate carrier, was also up-regulated (Barreteau et al., 2008; Lovering et al., 2012). Peptidoglycan is the main content of bacterial CW and plays crucial roles in keeping CW integrity and morphology (Typas et al., 2012). The CW was destroyed after treatment with linalool (Figure 2), thus we speculate the increase in peptidoglycan synthesis is the cell response to cellular damage. Increased peptidoglycan synthesis may be also associated with the presence of the mesosome-like structures.

Transport systems are all involved in the transport of materials into or out of the cell. ABC transporters transport a large variety of substrates, such as ions, sugars, lipids, proteins, peptides, sterols and drugs (Rees et al., 2009). In our study, the regulation of ABC transporters were shown in Supplementary Figure S2. *potA*, *potB*, *potC*, and *potD*, involving in the spermidine/putrescine transport system, were all down-regulated. PotA was membrane-associated protein, PotD was periplasmic substrate binding protein, PotB and PotC were transmembrane proteins forming channels for spermidine and putrescine, respectively. Spermidine and putrescine are the main component of polyamines, and the decrease in transport capacity of polyamines could influence cell proliferation and differentiation (Kashiwagi et al., 1995, 1996; Igarashi and Kashiwagi, 1999). Genes involving in glycine betaine (*proX*, *proW*, and *proV*) and osmoprotectant (*opuA*, *opuB*, and *opuC*) transport system were all up-regulated. The accumulation of glycine betaine and osmoprotectant could help to protect cells against osmotic stress (Ko et al., 1994; Ko and Smith, 1999). Genes related to iron complex (*fhuD*, *fhuB*) and iron (*mtsA*, *mtsB*, and *mtsC*) uptake were up-regulated. Iron mediates many cellular activities and is essential for the survival of bacteria (Raymond et al., 2003; Rouault, 2004). Hence, our finding indicated that cells experienced iron-limitation after linalool treatment, the increased uptake of iron is the cell response for survival. Meanwhile, genes involved in cation binding and metal ion binding were also significantly regulated, which also indicated that the concentration of some cations and metal ions may be significantly altered.

The phosphotransferase system (PTS) involves the uptake and phosphorylation of carbohydrates from the extracellular environment (Kotrba et al., 2001). In our study, *pts*I (enzyme I) and *crr* (sugar-specific IIA component) were up-regulated, they are essential for the uptake of carbohydrates of Glucose-Glucoside (Glc) family, such as glucose, maltose, α- and β- glucoside and trehalose (Tchieu et al., 2001) (Supplementary Figure S3). Cells may increase the uptake of nutrients (carbohydrate) from environment to gain more energy to repair damage caused by linalool. Almost all the DEGs related to ribosomes, RNA degradation, and DNA replication pathways were up-regulated (Supplementary Figures S4–S6). The bacterial ribosome is a cytoplasmic organelle regarded as the site of mRNA translation and protein synthesis, thus the up-regulation of these pathways implies an increase in protein synthesis. The ribosome and nucleoid could be potential intracellular targets of linalool. More research is needed to more fully explore the protein changes after treatment with linalool.

## Conclusion

In conclusion, our study demonstrated that linalool is an effective and promising anti-listeria agent against both planktonic cells and biofilms. To the best of our knowledge, this is the first study examining the effect of linalool on *L. monocytogenes* biofilm. Our study provides insights into the potential antimicrobial mechanism of linalool, however, further studies should be carried out to identify the exact mechanism of action. Moreover, we will extend our study to a broader set of *L*. *monocytogenes* strains, especially field strains isolated from food or food processing environments.

## Data Availability Statement

The datasets generated for this study can be found at NCBI, GSE136998.

## Author Contributions

All authors listed have made a substantial, direct and intellectual contribution to the work, and approved it for publication. ZG and JG conceived and designed the experiments. WZ and KC performed the experiments. ZG and JG analyzed the data and wrote the manuscript. JV and JZ revised the manuscript. All authors read and approved the final manuscript.

## Conflict of Interest

The authors declare that the research was conducted in the absence of any commercial or financial relationships that could be construed as a potential conflict of interest.

## Footnotes

1 https://www.who.int/news-room/fact-sheets/detail/listeriosis

2 https://pubchem.ncbi.nlm.nih.gov/compound/6549
