# Supplementary material for: Anti-listeria Activities of Linalool and Its Mechanism Revealed by Comparative Transcriptome Analysis
Source: Front Microbiol. 2019 Dec 20;10:2947. doi: 10.3389/fmicb.2019.02947 (PMC6938037; doi:10.3389/fmicb.2019.02947)
Supplement: Supplementary file 1 [file Data_Sheet_1.docx]

**Supplementary Figure Captions**

**Figure S1**. Peptidoglycan biosynthesis pathway from the KEGG annotation analysis. The genes with red/green borders belong to the differential genes detected by RNA- sequencing, in which red represents the up-regulated genes and green represents the down-regulated genes.

**Figure S2**. ABC transporter pathway from the KEGG annotation analysis. The genes with red/green borders belong to the differential genes detected by RNA- sequencing, in which red represents the up-regulated genes and green represents the down-regulated genes.

**Figure S3**. Phosphotransferase system (PTS) pathway from the KEGG annotation analysis. The genes with red/green borders belong to the differential genes detected by RNA- sequencing, in which red represents the up-regulated genes and green represents the down-regulated genes.

**Figure S4**. Ribosome pathway from the KEGG annotation analysis. The genes with red/green borders belong to the differential genes detected by RNA- sequencing, in which red represents the up-regulated genes and green represents the down-regulated genes.

**Figure S5**. RNA degradation pathway from the KEGG annotation analysis. The genes with red/green borders belong to the differential genes detected by RNA- sequencing, in which red represents the up-regulated genes and green represents the down-regulated genes.

**Figure S6**. DNA replication pathway from the KEGG annotation analysis. The genes with red/green borders belong to the differential genes detected by RNA- sequencing, in which red represents the up-regulated genes and green represents the down-regulated genes.

**Figure S1**

**
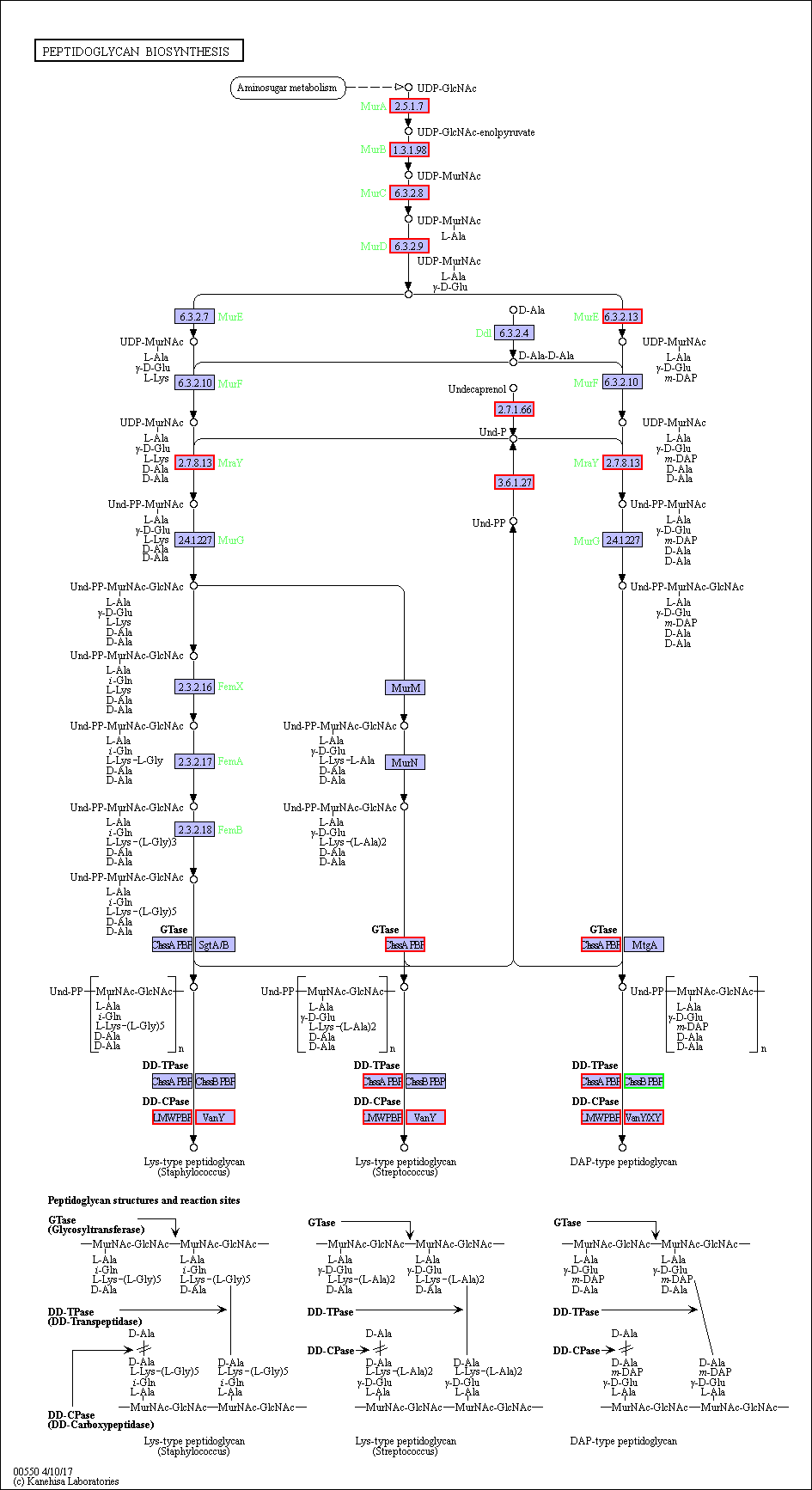
**

**Figure S2**

**
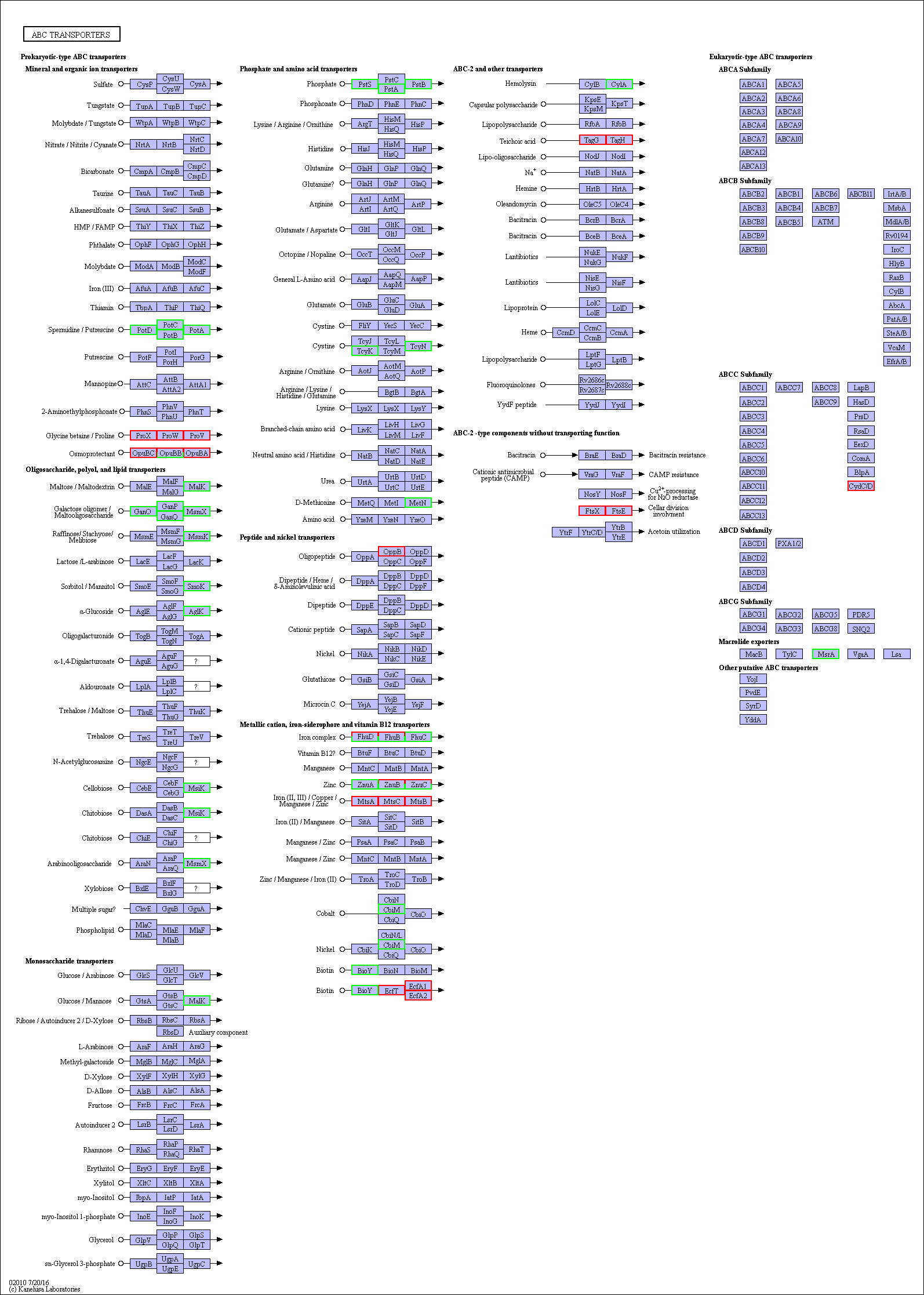
**

**Figure S3**

**
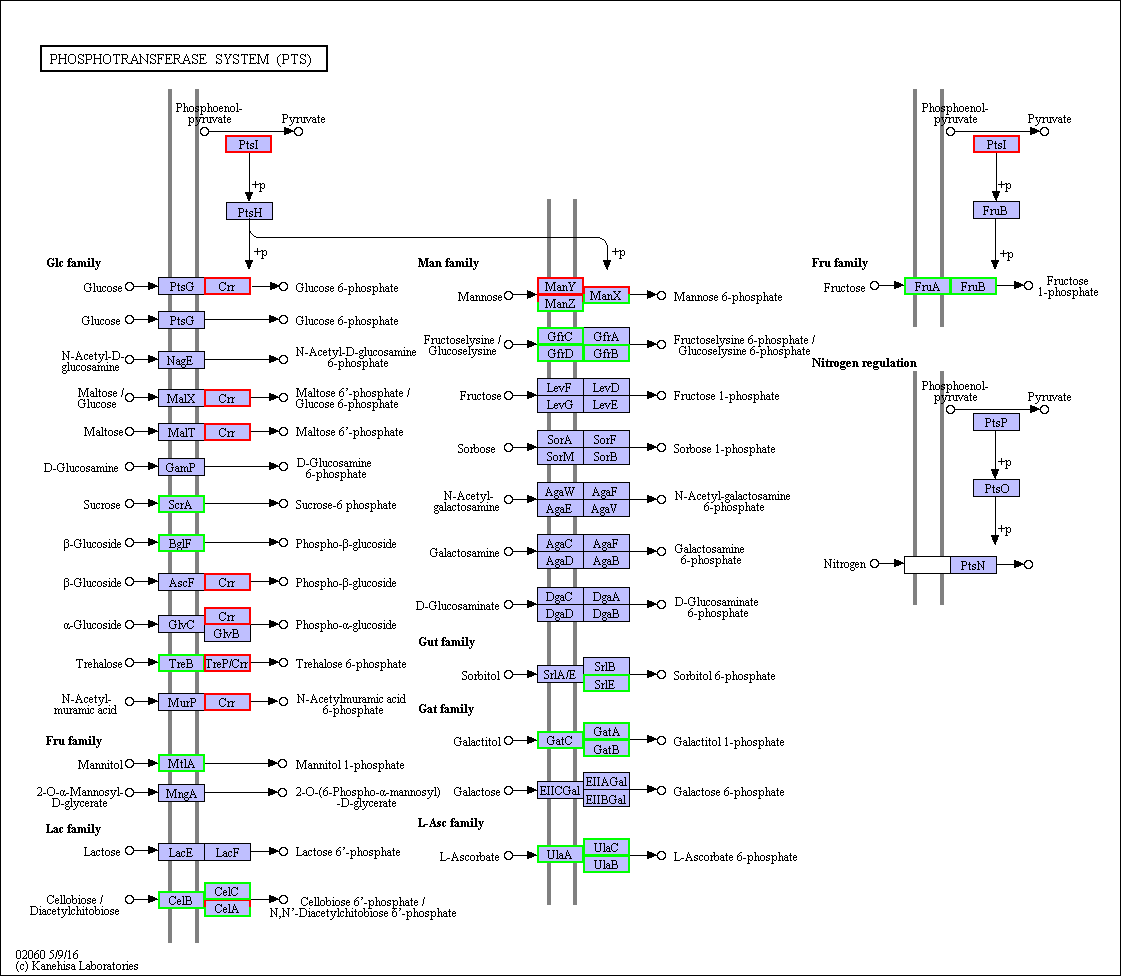
**

**Figure S4**

**
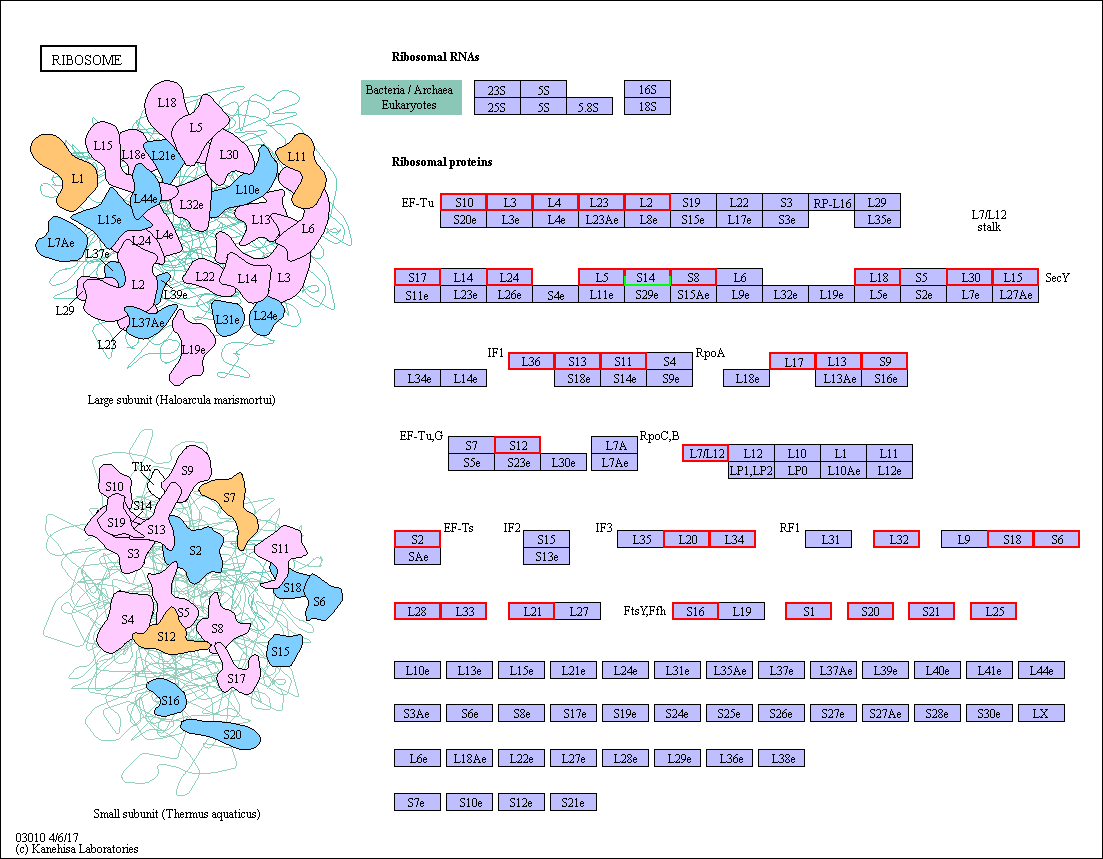
**

**Figure S5**

**
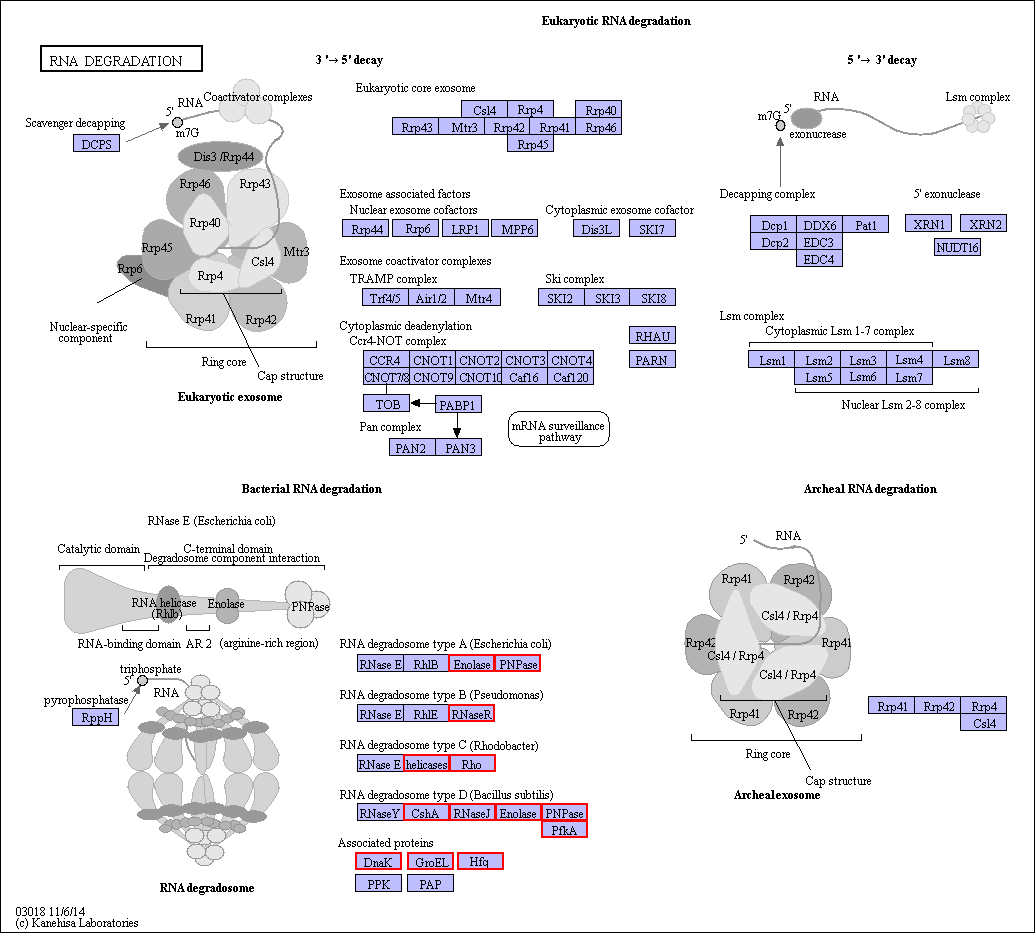
**

**Figure S6**

**
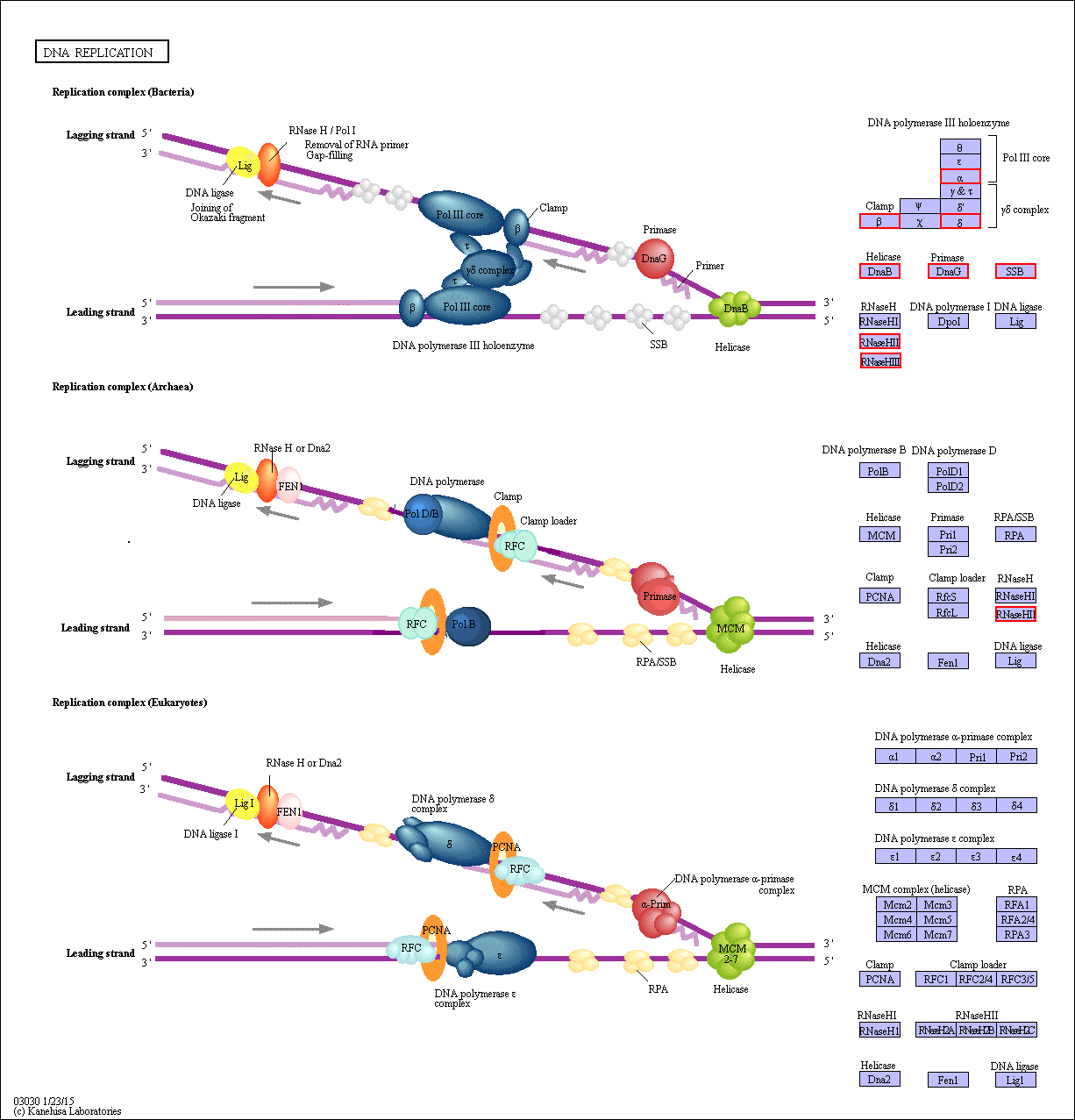
**
